# Social touch deprivation during the COVID-19 pandemic and reduced well-being

**DOI:** 10.3389/fpsyg.2025.1672502

**Published:** 2026-01-08

**Authors:** Antje B. M. Gerdes, Svenja Mareike Roether, Georg W. Alpers

**Affiliations:** 1 Department of Psychology, School of Social Sciences, University of Mannheim, Mannheim, Germany; 2 Otto Selz Institute, University of Mannheim, Mannheim, Germany

**Keywords:** social touch, well-being, resilience, COVID-19, social distancing

## Abstract

**Introduction:**

Social distancing was mandatory during the COVID-19 pandemic to curtail the spread of infections. This hampered interactions with people who did not share households, including the exchange of social touch. We explored how social distancing influenced people’s experience of social touch and its impact on psychological well-being during the pandemic.

**Methods:**

In an online survey carried out in Germany (*N* = 287; 77.5% female), we assessed the estimated number of episodes per day involving social touch in different contexts (personal and professional surroundings) as well as individual factors that may influence the frequency of social touch (e.g., relationship status, living arrangement). Participants retrospectively compared episodes for the time before the outbreak versus during mandatory social distancing. In addition, we examined whether social touch predicts psychological well-being and sought to identify protective factors.

**Results:**

As expected, during the pandemic, social touch was less frequent in both personal and professional settings. However, individuals in a relationship reported relatively stable levels of social touch in personal contexts, unlike those without a relationship. Overall well-being declined during the pandemic, but this decline was less pronounced among those who experienced more social touch independently of relationship status.

**Discussion:**

Social distancing in a Western society substantially reduced social touch, which in turn harmed psychological well-being, supporting the notion that touch is a relevant factor for resiliency. This research highlights that individuals who are not in a relationship are particularly vulnerable to the effects of social distancing.

## 1 Introduction

In order to control the spread of COVID-19, social distancing was imposed worldwide (see, e.g., Cohen and Kupferschmidt, 2020). The physical separation of individuals prohibited personal meetings, and social touch between individuals living in different households was severely restricted. Thus, this unfortunate situation provided a rare opportunity to investigate the consequences of reduced social touch on a large scale.

Social touch, including the exchange of touch between conspecifics, is an integral part of human nature and accompanies us throughout our lives (Cascio et al., 2019). During pregnancy, touch is the first sensory modality of social exchange a child develops (Bremner and Spence, 2017). Across the entire human lifespan, social touch serves as a fundamental organizing principle of development, influencing the formation of attachment, the experience of social reward, cognitive and communicative capacities, and the regulation of emotions from early infancy into adulthood and aging (see, e.g., Cascio et al., 2019; Ardiel and Rankin, 2010; Bellieni et al., 2007). Furthermore, touch becomes a crucial element in social interaction and interpersonal relationships (Field, 2010; Saluja et al., 2024).

Through touch, humans convey distinct emotions, secure the compliance of others, and reinforce bonds in romantic relationships (Chatel-Goldman et al., 2014; Gallace and Spence, 2010; Hertenstein et al., 2009). Although the concept of social touch appears to be universal, research has demonstrated that factors such as age, gender, context, and culture significantly influence how individuals touch each other and perceive touch (Gallace and Spence, 2010; Remland et al., 1995; Williams and Willis, 1978). Although the concept of social touch appears to be universal, research has shown that factors such as age, gender, context, and culture significantly influence how individuals touch and perceive touch (Gallace and Spence, 2010; Remland et al., 1995; Williams and Willis, 1978). For example, women tend to experience more positive touch during their life span than men (Webb and Peck, 2015), and the pleasantness of touch increases with age (Sehlstedt et al., 2016). Cultural influences are also evident in touch — touch is more frequent in Southern European countries than in Northern European countries (Remland et al., 1995) and Western individuals experience social touch as more pleasant than Asian individuals (Suvilehto et al., 2019).

Importantly, touch improves physical and mental health and well-being of human beings at various levels (Field, 2010). For example, manual massage therapy on preterm infants has led to reduced stress (Field et al., 2006b) and weight gain in preterm infants (Field et al., 2006a). Furthermore, longitudinal and experimental studies have shown positive effects of social touch on psychological well-being (e.g., Debrot et al., 2013) and physical well-being (e.g., due to lower blood pressure and higher oxytocin activity as physiological mediators between touch and well-being; Light et al., 2005). An intervention study with institutionalized elderly female residents showed that the group, which received daily comforting social touch in the form of handshakes or shoulder and arm patting during a 5-min social interaction, reported greater well-being measured by a multidimensional self-report mood inventory, health status, and life satisfaction at the end of a four-week period compared to the control group without the social touch intervention (Butts, 2001). On the other hand, touch deprivation can have dramatic and detrimental effects on the physical and psychological development of children, for example in form of persistently elevated stress levels (Nikolaeva et al., 2024) or delayed language development (Frank et al., 1996).

A model on the beneficial effects of affectionate social touch on well-being outlines three distinct but interconnected pathways (Jakubiak and Feeney, 2017). First, touch can reduce stress by modulating autonomic nervous system activity, lowering cortisol levels, and reducing physiological stress responses. Second, touch can influence cognitive-relational mechanisms: it serves as a nonverbal signal of support and affection, enhancing perceptions of relational quality, emotional security, and social belonging. Third, touch engages neural and neurobiological pathways, primarily via the release of oxytocin and endogenous opioids, which facilitate social bonding, trust, and positive affect (e.g., Morhenn et al., 2012). On a neural level, affective touch shifts the processing priority of the amygdala from exteroception to interoception (Gothard and Fuglevand, 2022). Taken together, this model integrates many observations on how affectionate social touch contributes to well-being through physiological, psychological, and neurochemical processes. Although this model focuses on affectionate touch, several studies indicate that any kind of non-aversive social touch fosters well-being even if it is not affectionate and even if it is exchanged among unrelated individuals (Lindgren et al., 2012). Furthermore, these positive influences are not limited to receiving social touch but are also beneficial for individuals who touch someone; they also experience reduced emotional stress and improved mental clarity (Debrot et al., 2013; Naruse et al., 2020).

### 1.1 Touch deprivation

While the positive effects of experiencing more social touch have been well-studied in several areas, the effects of touch deprivation are more difficult to assess.

Due to the difficulties and ethical dubiousness of implementing touch deprivation, findings on the effects of touch deprivation result from exceptional situations (e.g., foster care or orphanage rearing; MacLean, 2003; Nikolaeva et al., 2024; Field, 2010) or animal studies (see Ma et al., 2022; Pietropaolo et al., 2008). Harlow’s surrogate mother experiments with rhesus monkeys showed, for example, that the infants highly preferred the comfort and contact of a “cloth mother” provided over the nursing of a “wire mother” (Harlow and Zimmermann, 1958). The effects intensified in unfamiliar situations, posing a threat potential. The complete social isolation of the rhesus monkeys was associated with further negative behavioral consequences such as aggressiveness, refusal of food intake or autistic self-clutching (Harlow et al., 1965). Moreover, a study with neglected orphans demonstrated how infants who only receive minimal comforting touch from their caregivers, later struggle with severe cognitive and neurodevelopmental delay (MacLean, 2003; Field, 2010; Blackwell, 2020).

Although generalizability of these studies is limited, findings from the previous COVID-19 pandemic indicate that persistent deprivation of social touch has the potential to impact an individual’s behavior and well-being—mainly due to the distressing nature of the pandemic itself (Siebenhaar et al., 2020; Durkin et al., 2021). Indeed, in recent studies during COVID-19, up to 60% of respondents stated feeling touch deprived during COVID-19. Thereby, feeling touch deprived was related to feelings of depression, anxiety, stress, and sleep disturbances (Field et al., 2020a, 2020b; Bruno et al., 2023) and longing for touch was associated with a lower physical, psychological and social quality of life (Hasenack et al., 2023). Similarly, it was shown that touch deprivation during COVID-19-related restrictions was associated with higher anxiety and greater loneliness (Mohr et al., 2021). However, another recent study on social touch, focusing only on married or romantic partners (Burleson et al., 2022), shows that physical distancing was associated with less psychological distress. Moreover, the relationship between physical distancing and affectionate touch seemed to depend on individuals’ living arrangements. More physical distancing was associated with less affectionate touch in individuals living alone but with more touch in individuals living with their partner.

Against the background of social distancing during the COVID-19 pandemic, the present study aims to further explore the role of social touch experiences (following the definition of social touch as intentional physical contact between individuals see, e.g., Suvilehto et al., 2023). In contrast to most of existing studies investigating touch through the pandemic (for an exception see Burleson et al., 2022), we intended to focus not only on the feeling of touch deprivation but on the estimated amount of social touch, using current and retrospective numerical data, in addition to the previously studied subjective perception of feeling touch deprived (e.g., Field et al., 2020a, 2020b; Burleson et al., 2022). Specifically, we examined whether social distancing limited the amount of experienced social touch, and which factors might protect an individual from such decline. We expected to observe a decline in the estimated frequency of social touch during the COVID-19 pandemic, as physical distancing measures and social restrictions limited opportunities for interpersonal contact. Moreover, given that relationship status and living arrangements have previously been associated with experiences of touch deprivation and reduced physical contact (Field et al., 2020b; Burleson et al., 2022), we hypothesized that this decline would be particularly pronounced among individuals living alone or without a relationship. This assumption is referred to as the *Reduction Hypothesis*, which predicts that the absence of close social partners amplifies the decrease in touch frequency during periods of social isolation (e.g., Burleson et al., 2022).

In a second step, we aimed to further examine the association between social touch and psychological well-being. Building on previous findings that emotional touch deprivation is associated with elevated stress and anxiety (Field et al., 2020a, 2020b), we sought to replicate these results and test the presumed negative effect of reduced touch on well-being. In addition, we investigated whether the estimated amount of social touch could predict individual differences in well-being both before and during the pandemic. In line with the notion that social touch promotes psychological and physical well-being (Jakubiak and Feeney, 2017), we formulated the *Well-being Hypothesis*, positing that higher estimates of social touch are associated with better psychological well-being.

## 2 Method

### 2.1 Participants

Participants were recruited in Germany through press reports, mailing, and posts on social media platforms. Participation was open to adults aged 18 years and older. Ethical approval was granted by the ethics committee of the University of Mannheim (EK Mannheim 47/2020). Exclusions occurred in cases of incomplete data sets by discontinuation of the study or unnaturally fast completion of the questionnaire as indicated by the speed index of SoSci Survey. This resulted in a final sample of *N* = 287 participants in Germany, of whom 77.5% were female and aged between 18 and 71 (*M* = 31.73, *SD* = 14.43). Furthermore, 55.1% of the participants were in a relationship, and 80.5% shared their home with at least one other person. A detailed description can be found in Table 1.

**Table 1:** Demographic overview separated by gender.

Variable	Mean	SD	Min	Max
Age				
Male	31.43	15.68	18	70
Female	31.64	13.93	18	71

Variables	Male		Female	
	*N*	%	*n*	%
Relationship status				
Single	33	50.8	85	38.3
Divorced	1	1.5	9	4.1
Married	6	9.2	43	19.4
In a relationship	21	32.3	71	32.0
In a long-distance relationship	4	6.2	13	5.9
In total without relationship	34	52.3	95	42.8
In total relationship	31	47.7	127	57.2
Living arrangement*				
Living alone	9	13.8	47	21.2
With a partner	22	33.8	78	35.1
With children	5	7.7	27	12.2
With parents	24	36.9	73	32.9
With siblings	16	24.6	43	19.4
Other family members	3	4.6	8	3.6
Other apartment sharing	13	20	28	12.6
In total living alone	9	13.8	47	21.2
In total living with others	56	86.2	175	87.8
Highest educational level				
Pupil	0	0	2	0.9
Secondary school diploma	4	6.2	11	5.0
Abitur	35	53.8	108	48.6
In training	0	0	7	3.2
College / University degree	26	40.0	91	41.0
Miscellaneous	0	0	3	1.4
Economic situation during COVID-19				
Extremely worsened	5	7.7	4	1.8
Worsened	10	15.4	49	22.1
Unchanged	49	75.4	161	72.5
Improved	1	1.5	6	2.7
Extremely improved	0	0	2	0.9

*Multiple selection.

### 2.2 Procedure

Data was collected from the middle to the end of the first so called “lockdown” period in Germany (April 9 until May 26, 2020). During that time, businesses, restaurants, and schools were closed in most parts of Germany, and the German government had imposed contact restrictions (Schilling et al., 2021). Accordingly, all questionnaires were completed online using SoSci Survey hosted on the University’s secure server. After reading a brief description of the study and its general objectives, participants gave their consent and provided their demographic information. Afterward, they responded to the questionnaires, which took approximately 40 min to complete. At the end of the study, participants could enter a lottery for shopping vouchers or, if they were University of Mannheim students, receive course credit.

### 2.3 Measures

#### 2.3.1 Demographic information

Participants were asked to indicate their age, gender, relationship status, educational background, work circumstances, income, and living arrangement (living alone vs. living together with someone such as a partner, parents, or flat mates). They were also asked to answer COVID-19-specific questions such as belonging to risk groups, a diagnosed or suspected infection, and quarantine measures.

#### 2.3.2 Well-being

Well-being and the quality of life were assessed with the EUROHIS-QOL and WHO-5 well-being index (Brähler et al., 2007). While the WHO-5 well-being index can be used as a screening instrument for Major Depression, the EUROHIS-QOL indicates the cross-sectional quality of life, including a psychological, physical, social, and environmental facet (Brähler et al., 2007). Due to the broader range of facets and the good internal consistency (Cronbach’s *α* = 0.85) and test–retest reliability according to Guttman (r_TT_ = 0.77), the EUROHIS-QOL was chosen as the primary outcome measure for general well-being (Brähler et al., 2007). In the present study, the EUROHIS-QOL was presented to the participants twice. First, participants were asked to rate their well-being during mandatory social distancing on a 5-point scale, ranging from 1 “very poor” to 5 “very well.” In addition, participants retrospectively evaluated their well-being before the pandemic using the same eight items.

#### 2.3.3 Social touch and social contact

To quantify the number of touches, participants estimated the number of experienced social touches on an average day during mandatory social distancing and retrospectively before the pandemic. Moreover, personal and professional contexts were evaluated separately. Participants also indicated how many direct (in-person meetings) and indirect (e.g., via phone) contacts they had since the COVID-19 pandemic started. Again, a distinction was made between personal and professional contexts.

Deprivation of touch was measured by asking the participants to rate their agreement to the statement “I miss physical contact (e.g., hugging) with other people” on a 5-point scale, ranging from 1 “not at all” to 5 “applies completely.” Behavior and attitudes towards social touch were examined using a German version of the Social Touch Questionnaire (STQ; Wilhelm et al., 2001; Vieira et al., 2016).

#### 2.3.4 Change in social behavior

The survey included three additional scales. On a fully labeled 3-point scale, ranging from 1 “less often than before” to 3 “more often than before,” participants rated how they had adapted their behaviors (e.g., “use of public transport”) and how their feelings (e.g., “feelings of loneliness”) had changed since the beginning of the COVID-19 pandemic. Participants could also indicate when they had never engaged in the behavior in question before or during COVID-19.

### 2.4 Statistical analysis

Statistical analyses were run in IBM SPSS Statistics (Version 21) and R (R Core Team, 2020). Prior to all analyses, the underlying assumptions were tested. All applied correction measures are reported below. An alpha level of 5% was used for all statistical analyses.

#### 2.4.1 Reduction hypothesis

First, we inspected the distributional properties of our primary outcomes. The estimates of social touch were positively skewed and, therefore, log-transformed to approach a more normal distribution. The estimates of social touch were compared between male and female participants. Since no relevant differences were found, the following analyses were performed without differentiation by sex. To test the *reduction hypotheses*, a mixed ANOVA with repeated measures was conducted. The 2 × 2 × 2 × 2 ANOVA incorporated the within-subjects factors of *time* (before the COVID-19 outbreak vs. during the COVID-19 pandemic) and *context* of touch (personal vs. professional), as well as the between-subjects factors *relationship status* (without vs. in a relationship), and living arrangement (living alone vs. together with others).

#### 2.4.2 Well-being hypothesis

The impact of touch and touch deprivation on well-being was examined by using separate linear mixed models (LMM). LMM was chosen since the observations of well-being, social touch, and touch deprivation were nested within participants. The aim was to examine whether the amount of experienced social touch and the feeling of touch deprivation can explain well-being and whether the nested data structure accounted for it. In addition, the effects of relationship status and context will be tested exploratively. The models were estimated in R (R Core Team, 2020) using the package lme4 (Bates et al., 2015).

#### 2.4.3 Touch deprivation

Finally, exploratory correlational analyses were used to examine the relationship between the feeling of being touch-deprived and the estimates of social touch. For this purpose, perceived touch deprivation was correlated with the estimates of social touch before and during the pandemic and with the difference score of social touch (Difference Score = Touch before COVID-19 – Touch during COVID-19).

## 3 Results

### 3.1 Descriptive data on experienced social touch

In the personal context, participants reported having experienced, on average, *M* = 24.09 (*SD* = 52.31, ranging from 0 to 700; *M*_log_ = 2.478) social touches per day before the pandemic. During the pandemic, estimates of social touch dropped to *M* = 16.62 (*SD* = 44.00, ranging from 0 to 400; *M*_log_ = 1.702). In the professional context, lower estimates of experienced social touch were reported, with *M* = 13.34 (*SD* = 23.21, ranging from 0 to 200; *M*_log_ = 1.9) before the pandemic and *M* = 0.55 (*SD* = 4.26, ranging from 0 to 60; *M*_log_ = 0.1) during the pandemic. An overview of statistical values can be found in Table 2.

**Table 2:** Correlational analyses of well-being, experienced social touch, and social contact.

Variables	*N*	*M*	*SD*	(1)	(2)	(3)	(4)	(5)	(6)	(7)	(8)	(9)
Well-being before the pandemic	287	31.87	4.68									
Well-being during the pandemic	287	30.16	4.99	0.694^**^	—							
Touch before the pandemic in personal	281	24.09	52.31	0.123^*^	0.072	—						
Touch during the pandemic in personal	281	16.62	44.0	0.202^**^	0.205^**^	0.695^**^	—					
Touch before the pandemic professional	282	13.34	23.21	0.032	−0.059	0.471^**^	0.327^**^	—				
Touch during the pandemic professional	282	0.55	4.26	−0.037	−0.024	0.206^**^	0.187^**^	0.233^**^	—			
Contact direct personal	287	2.3	1.82	0.01	0.045	0.214^**^	0.256^**^	0.12^*^	0.075	—		
Contact direct professional	287	2.47	8.35	−0.05	0.18	0.065	0.045	0.172^**^	0.035	0.113	—	
Contact indirect personal	287	5.29	4.28	0.1	0.019	0.171^**^	0.092	0.134^*^	0.012	0.177^**^	0.001	—
Contact indirect professional	287	4.53	7.36	0.151^*^	0.179^**^	0.145^*^	0.172^**^	0.211^**^	0.0	0.031	0.436^**^	0.052

*p < 0.05, two-tailed **p < 0.01, two-tailed. Means and standard deviations of social touch and social contact refer to the original values before the transformations. The data on social contact refers only to the period during the pandemic living.

### 3.2 Decrease of social touch due to social distancing

To test the assumptions of the *reduction hypothesis* a 2 × 2 × 2 × 2 mixed ANOVA, with repeated measures was conducted. Beginning with the significant interaction effect, analyses showed a statistically significant three-fold interaction between time, context, and relationship status *F*(1, 274) = 7.12, *p* = 0.008, partial *η^2^* = 0.03 (a complete overview of all main and interaction effects, including the two-fold interactions, can be found in Table 3). The four-fold interaction between time, context, relationship status and living arrangement was not statistically significant, *F*(1, 274) = 0.01 *p* = 0.943, partial *η^2^* < 0.001. Similarly, the further three-fold interactions between time, relationship status, and living arrangement, *F*(1, 274) < 0.01, *p* = 0.965, partial *η^2^* < 0.001, the interaction between context, relationship status, and living arrangement, *F*(1, 274) = 0.24, *p* = 0.625, partial *η^2^* < 0.001, as well as the interaction between time, context, and living arrangement, *F*(1, 274) = 2.62, *p* = 0.106, partial *η^2^* = 0.01, were not statistically significant. Due to the significant three-fold interaction, significant two-fold interactions and main effects were not interpreted.

**Table 3:** Results of a mixed ANOVA with repeated measures on the log-transformed outcome social touch.

Effect	*SS*	*Df*	*F*	*p*	*η^2^_p_*
Time	234.255	1	346.724	<0.001	0.559
Context	86.007	1	96.394	<0.001	0.260
Two-fold interactions					
Time × Relationship status	4.888	1	7.235	0.008	0.026
Time × Living arrangement	0.168	1	0.249	0.618	0.001
Time × Context	24.671	1	43.862	<0.001	0.138
Context × Relationship status	16.658	1	18–655	<0.001	0.064
Context × Living arrangement	25.796	1	28.888	<0.001	0.095
Three-fold interactions					
Time × Relationship status × Living arrangement	0.001	1	0.002	0.965	0.000
Time × Context × Relationship status	4.006	1	0.7.122	0.008	0.025
Time × Context × Living arrangement	1.467	1	2.624	0.106	0.009
Context × Relationship status × Living arrangement	0.214	1	0.240	0.625	0.001
Four-fold interaction					
Time × Context × Relationship status × Living arrangement	0.003	1	0.005	0.943	<0.001

Following the statistically significant interaction between time, context, and relationship status, the effects were further analyzed by splitting the data according to the factor context. For personal contexts, analyses showed a significant main effect of time *F*(1,279) = 196.19; *p* < = 0.001; partial *η^2^* = 0.413, as well as a significant interaction of time and relationship status *F*(1,279) = 44.66; *p* < = 0.001; partial *η^2^* = 0.138 indicating that individuals with a relationship had experienced significant more social touch than individuals without a relationship in personal contexts before and even more pronounced during the pandemic, [Before COVID-19 *M*_Relationship_log_ = 2.69, *SD* = 1.13; *M*_WithoutRelationship_log_ = 2.22, *SD* = 1.17; *t*(279) = 3.44; *p* < =0.001; during COVID-19 *M*_Relationship_log_ = 2.26, *SD* = 1.34; *M*_WithoutRelationship_log_ = 1.01, *SD* = 1.24; *t*(279) = 8.08; *p* < = 0.001]. For the professional context, there was also a main effect of time [*F*(1,278) = 578.7, *p* = 0.001, eta = 0.676], but in contrast to the personal context, no significant interaction between relationship status and time [F(1,278) = 0.006, *p* = 0.939, eta<= 0.001; Before COVID-19 *M*_Relationship_log_ = 1.90, *SD* = 1.21; *M*_WithoutRelationship_log_ = 1.90, *SD* = 1.32; t(280) = 0.046; *p* = 0.963; during COVID-19 *M*_Relationship_log_ = 0.10, *SD* = 0.48; *M*_WithoutRelationship_ = 0.10, *SD* = 0.47; *t*(280) = 0.10; *p* = 0.921]. These results show that the relationship status of an individual influenced the estimates of experienced social touches in personal but not in professional surroundings. In the personal context, individuals in a relationship estimate more social touches than individuals with no relationship, especially during the pandemic.

**Figure 1:**
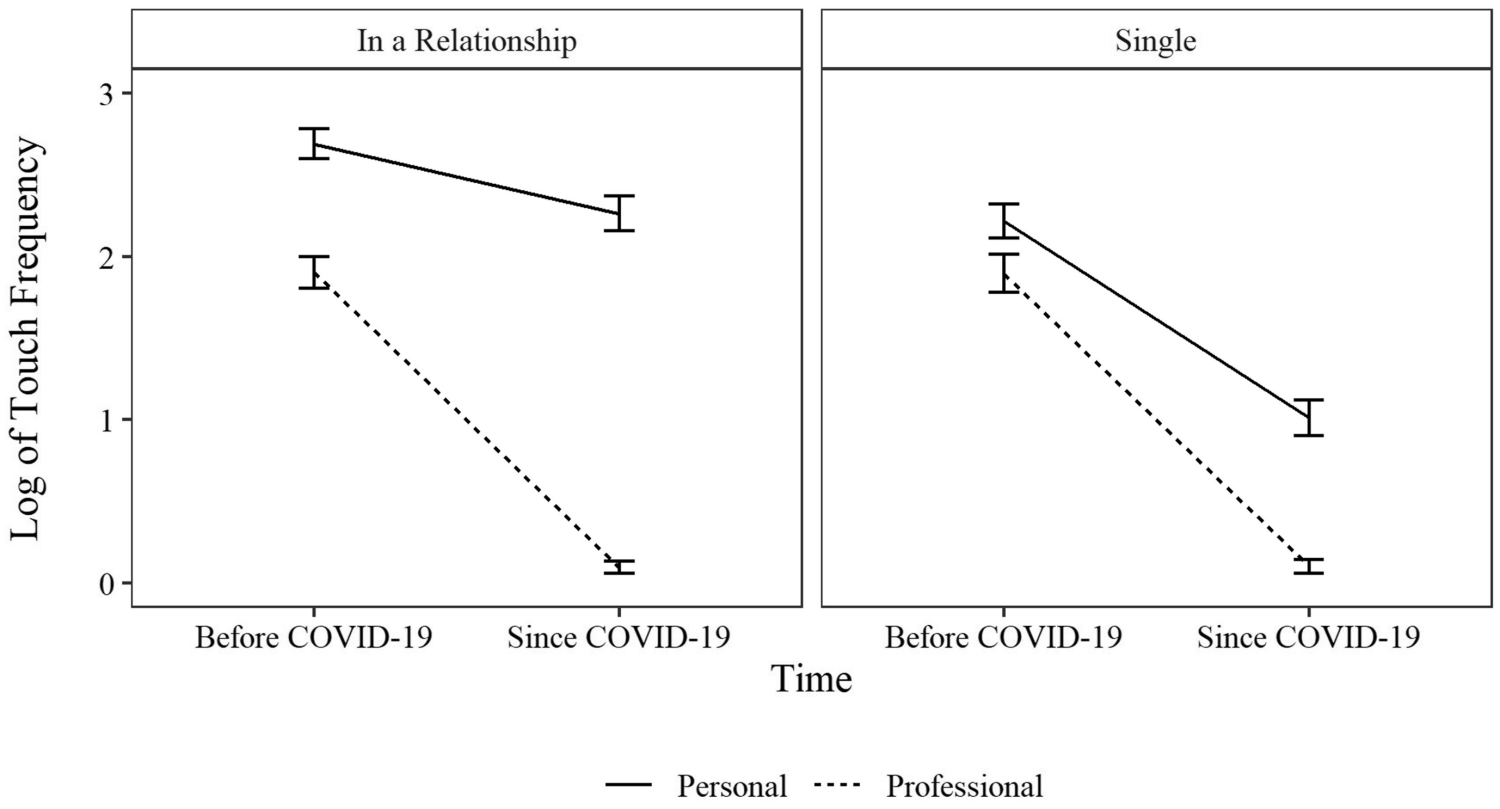
Log-transformed means and standard errors of the amount of social touch at different points in time separately for personal and professional contexts. Separate display for individuals in and without a relationship.

Taken together, the statistically significant interaction between time, context, and relationship status indicated that in a personal context, individuals who were in a relationship were posing an exception to the overall steep decline in social touch (Figure 1). Therefore, being in a relationship buffered the restraining effects of social distancing on the amount of experienced social touch in personal surroundings. Although sharing a household with others was associated with more experiences of social touch, the non-significant three-fold interaction between time, context, and living arrangement indicated that sharing a household did not have the same buffering effect as being in a relationship. Thus, the assumptions of the reduction hypothesis can be partially confirmed.

### 3.3 Regression of well-being

To test the *well-being hypothesis*, linear mixed models were specified, including the estimates of touch and time as well as touch deprivation and time. A random intercept model was specified to test whether it is meaningful to account for the nesting of observations within individuals. By clustering observations within subjects alone, 77% of the variance in the well-being variable was between clusters and could be explained. However, 23% of the variance was located within individuals.

### 3.4 Estimates of social touch

Adding touch to the model equation yielded a better model, indicated by a significant Chi^2^ difference test, χ^2^(1) = 69.23, *p* < 0.001. Next, the model was extended by adding the variable time, χ^2^(1) = 96.00, *p* < 0.001. However, the interaction between both terms did not improve the model fit, χ^2^(1) = 1.96, *p* = 0.162, indicating that the influence of social touch on well-being did not differentiate over time. Therefore, well-being was best explained by a model that included time and social touch.

In the final model, 80.999% of the variance in well-being was explained. 3.136% of the variance was due to the fixed effects of social touch and time. The regression coefficients indicated that experiencing more touch was associated with higher well-being, *β* = 0.135; *t* (906.65) = 2.15, *p* = 0.032. Moreover, they indicated a lower well-being during the pandemic, *β* = −1.522; *t* (860.453) = −10.1, *p* < 0.001.

### 3.5 Explorative analysis

Exploratory analyses showed that adding context (personal/professional) as a categorical predictor variable did not increase the model fit compared to the final model containing social touch and time as predictors, χ^2^(1) = 2.14, *p* = 0.144. However, adding the relationship status did, χ^2^(1) = 17.74, *p* < 0.001. In the latter model, 81.02% of the variance was explained in total. Of that, 3.23% was due to the fixed effects. There was a significant prediction of well-being by time, *β* = −1.538; *t* (860.351) = −10.206, *p* < 0.001, and the relationship status of individuals, *β* = −2.203; *t* (285.458) = −4.278, *p* < 0.001. The latter coefficient implies lower well-being for individuals without a relationship. However, the effect of touch was not significant in this model, *β* = 0.122; *t* (906.05) = 1.94, *p* = 0.052. Thus, there was no effect of touch on well-being when controlled for relationship status.

#### 3.5.1 Touch deprivation

Correlational analyses showed that well-being during the pandemic correlated significantly negative with touch deprivation, *r* = −0.19, *p* < 0.001, indicating a lower sense of well-being in the case of pronounced touch deprivation. A separate analysis by relationship status showed that the negative correlation is mainly due to individuals in a relationship, *r* = −0.17, *p* = 0.002. Within the group of individuals without a relationship, the correlation from touch deprivation and well-being during the Covid-19 pandemic was no longer significant, *r* = −0.09, *p* = 0.16.

Starting from the random intercept model, the inclusion of touch deprivation did not improve the model equation as indicated by the likelihood-ratio test, *χ*^2^(1) = 2.86, *p* = 0.09. Nevertheless, the predictor was kept in case of interactions. Extending the model by adding the variable time and its interaction with touch deprivation yielded an improvement, *χ*^2^(3) = 227.88, *p* < 0.001. The regression coefficients showed a significant negative interaction between time touch deprivation, *β* = −0.762; *t* (842.273) = −8.183, *p* < 0.001, indicating a buffering of time on touch deprivation.

#### 3.5.2 Association of feeling touch deprived and estimates of social touch

Explorative analyses revealed a significant negative correlation between missing social touch and estimates of social touch during the pandemic in personal contexts, Spearman’s *ρ* = −0.183, *p* < 0.001, indicating a higher craving for social touch among those who estimate less of it. Furthermore, the changes in the experience of social touch were moderately positively correlated with the feeling of missing social touch, Spearman’s *ρ* = 0.324, *p* < 0.001. This indicates that the greater the difference in the estimates of touches during the pandemic and before, the more people missed these touches. This does not only apply to the personal setting but can also be found in a weakened form in the professional setting, Spearman’s *ρ* = 0.173, *p* < 0.001.

Explorative analyses showed a significant negative correlation between the estimated amount of received social touch and the feeling of touch deprivation during the pandemic, Pearson’s product–moment correlation *r* = −0.1, *p* < 0.017, indicating that the more social touch a person estimates, the less deprived they are. Due to the low correlation, it can be assumed that touch and touch deprivation have a common component but measure other aspects separately.

## 4 Discussion

Social distancing during the COVID-19 pandemic was a harsh measure devised to curb infections (Naumann et al., 2020). Our survey documents how this has influenced the experience of social touch and its impact on psychological well-being. For this purpose, participants estimated how often they experience social touch on average per day in different contexts and retrospectively how it was before the pandemic. As expected, the study results indicate that the COVID-19 restrictions have led to a decrease in daily experienced social touch. However, being in a relationship had a buffering effect on the decline of the experienced social touches in the personal contexts of an individual. Sharing a household with others does not protect against the decline due to social distancing (reduction hypothesis). Furthermore, the amount of experienced social touch predicts an individual’s well-being: the more daily touches an individual experiences, the better. While well-being decreased markedly during COVID-19, the importance of social touch for well-being has not changed as a result of the pandemic (well-being hypothesis).

### 4.1 Social distancing and social touch

The observed general decline of social touch due to social distancing is in line with our expectations as well as with the results of earlier studies on the consequences of the COVID-19 pandemic (e.g., von Mohr et al., 2021; Zhang et al., 2021). However, differences in living factors are critical in determining how much following the distancing rules affects an individual’s daily experience of social touch. In contrast to existing studies, we decided to focus on social touch in different contexts (personal vs. professional) and on interpersonal factors (living arrangement and relationship status). This approach allowed us to investigate specific contexts and factors that contribute to touch decrease and deprivation. Identifying those protective factors facilitates recognizing and providing compensation to those most vulnerable. It has already been shown that physical distancing leads to an increase in affectionate touch among cohabiting couples (Burleson et al., 2022). Thus, it is in line with our expectations that being in a relationship has a buffering effect on decreasing social touch in the personal context. Contrary to our expectations, sharing a household with others did not have the expected buffering effect on the measures of social distancing. In Germany, 20% of the population lives in single-person households. However, this is particularly true for older women and young men (Ditzen et al., 2021). Both groups, young men and older women, are underrepresented in the sample on which our study is based. Therefore, further studies that differentiate according to sex and age are needed. However, the importance of relationship status was already demonstrated. Between the ages 20 to 29 and in the age group over 70 years, the proportion of individuals who are not in a relationship is highest (19% and 22% respectively; Statistica, 2021). In our study, even more, i.e., 49% are single in the same young age group, which underlines the importance of accounting for relationship status. While being in a relationship and sharing a household with others is certainly confounded, especially in married individuals or individuals in a permanent relationship, it is not necessarily to be differentiated in a younger sample as in this study. In the present study, most of those who share a household with others share it with roommates, parents, or other family members such as siblings. Thus, they might not exchange as many touches with family members or friends as cohabiting couples would do, which may be a possible explanation for the missing buffering main effect of sharing a household.

Regarding the buffering effect of a relationship, it would be interesting to determine the particular factors in a relationship that cause individuals with a partner to experience more daily touches. However, the underlying data of our study does not allow for specific types of touch (e.g., whether partners exchange more affective touch in the form of intimacy or whether frequency and regularity are the keys). Thus, further research is needed to identify possible influencing factors based on the present study’s results.

Our results differ from the findings of Burleson et al. (2022), who found that living arrangements were linked to the amount of affectionate touch - for individuals who live in a relationship. However, in our study, cohabitation includes not only partners. Thus, the studies provide information about different groups of people and circumstances. Future studies should examine these distinctions more closely.

### 4.2 Social touch and well-being

Our study’s results support and extend existing literature regarding social touch and touch-deprivation effects on an individual’s psychological well-being. In line with the results of earlier studies on touch deprivation during the pandemic, the results of the present study show that the pandemic has resulted in a deterioration of well-being and that the experience of social touch positively influences an individual’s well-being (e.g., Field et al., 2020b; von Mohr et al., 2021). Thereby, social touch has been important for an individual’s well-being before and during the pandemic and did not change due to the pandemic (Dagnino-Subiabre, 2022). Furthermore, our exploratory results revealed that the effect of touch vanishes when controlling for the relationship status of an individual. This suggests that being in a relationship explains more well-being variance than social touch alone, indicating that although touch plays an important role, there are more aspects in social interaction that contribute to increased well-being. In order to determine the particular factors in a relationship that improve well-being and to rule out potential moderating and mediating effects (e.g., the personal need for touch), further research is needed.

In addition, the present research further extends existing research by introducing a new approach. In addition to assessing touch deprivation by directly asking participants how touch-deprived they feel (e.g., Field et al., 2020a, 2020b), we also chose a more objective approach by asking participants to express the frequencies of social touch in numbers. Explorative analyses revealed that these two concepts, the feeling of touch deprivation and the numeric amount of and changes in social touch, are correlated as expected. Individuals who experience little social touch and individuals who experience the most significant changes, independent of the baseline level, indicate that they miss social touch the most. The moderately high correlation underlines the plausibility of the data and suggests that while both constructs represent a common core, each variable contributes its part. Nevertheless, the following factors must be considered: First, although the numeric approach is a less direct approach to touch deprivation, the data still originated from the participants’ subjective and partly retrospective recall. Second, how much or little touch is perceived as deprivation depends on interindividual factors, such as liking and need for touch or personal experiences. This is further complicated by the difficulty in measuring everyday touches outside of laboratory experiments or through ambulatory assessments. To the best of our knowledge, no studies have been published in recent years that provide accurate information on the average frequency of social touch based on observations or other methods. Studies that counted naturally occurring touch for cultural comparisons only reported it as a percentage (Remland et al., 1995). Only one empirical overview, which included data from several observational studies, provided detailed information on social touch per minute and hour (Stier and Hall, 1984). Hence, we had to rely on the range of values given in the overview and plausibility checks to examine the validity of the present study’s data.

Nevertheless, further research to assess the relationship between feelings of touch deprivation and touch frequency and the potential differential influence of these two factors on well-being is highly needed.

### 4.3 Limitations

Although the COVID-19 pandemic provided a rare opportunity to investigate the relevance of social touch under the influence of official social distancing measures, the rapidly changing pandemic situation also required immediate data collection. While instant data collection was necessary, it also came at the cost of several limitations, under which our findings must be considered. First, the online data collection yielded a sample primarily consisting of young, female, and highly educated individuals from Germany. The non-representativeness of our sample reflects the current state in research on social touch (Mohr et al., 2021; Field et al., 2020b). Particularly in the research area on social touch, a non-representative sample could be seen as problematic due to sex, age and cultural differences in touch behavior (Remland et al., 1995). Since there were no relevant sex-specific group differences in touching behavior and demographic variables in the present sample, we decided not to differentiate by sex. Nevertheless, future studies with representative samples and balanced sex ratios would be desirable.

Furthermore, future studies should investigate the role of social touch in mental disorders (Müller-Oerlinghausen and Eggart, 2021). In this context, a small number of findings from clinical samples suggest, for example, that the need for—and appraisal of—social touch may differ in clinical populations such as individuals with borderline personality disorder (Schulze et al., 2022) or autism spectrum disorder (Fukuoka et al., 2025). Moreover, it can be assumed that social touch also holds therapeutic potential in the context of mental disorders (Papi et al., 2025; Packheiser et al., 2024).

Another limitation concerns the inherently inseparable interplay between social contact and social touch. Consequently, potential improvements in well-being may stem not only from the touch itself but also from the broader psychosocial context of interacting with others—such as increased social support (Reddan et al., 2020). Previous studies have addressed this methodological challenge by experimentally isolating touch from mere social presence and were thus able to demonstrate the unique beneficial effects of social touch beyond social contact (e.g., Butts, 2001). However, in the present study, such a distinction is not possible due to the naturalistic context of data collection. Furthermore, our findings indicate that social touch and social contact are positively correlated, suggesting that individuals who engage in more frequent or intensive interpersonal contact also tend to experience higher levels of social touch. This association complicates causal inferences about whether improvements in well-being are primarily attributable to the touch experience itself or to the social interaction it entails. However, the low to medium association in our data suggests a common core, with both variables contributing separately. However, since the data on social contacts refers only to the period during the COVID-19 pandemic, the correlation between social touch and social contact might be influenced by two important aspects: First, due to the pandemic and restrictive regulations, many social touches of the participants could result from their household (e.g., family members or roommates), which may not have been perceived as each being an independent social contact. In addition, the fear of infection during the pandemic has led to social contact and touch being perceived as negative and harmful (Ditzen et al., 2021; Ko et al., 2020). Therefore, we decided not to include social contact as a moderating factor. Since the additional impact of social contact cannot be ruled out in our study, future studies should include this aspect to study the effects of social touch more isolated.

In addition, as we found that the impact of social distancing differs for individuals in different living arrangements or for people with different needs, our findings correspond with other observations that personal relevance needs to be assessed in COVID-19-related decision-making (Köther et al., 2021).

Furthermore, the study results must be considered in light of the shortcomings of the study design. Since we conducted a cross-sectional survey with a longitudinal design approach, the causal inferences drawn from our data are partly limited. The study was initiated with the outbreak of the COVID-19 pandemic; thus, no data on social touch and well-being before the pandemic was available. Whereas the retrospective survey approach allowed longitudinal comparisons, it is also a relevant limitation of our study that the number of episodes with social touch was captured via retrospective self-report, which may be influenced by memory biases as well as emotional and motivational states (Coughlin, 1990). However, this is not uncommon in established self-report measures of touch (e.g., TEAQ or TEAQ-s). These scales have good internal consistency and construct validity (Trotter et al., 2018; Friedrich et al., 2025). Nonetheless, our findings should be interpreted with caution, and future studies should include real-time assessments or sensors data (e.g., continuous measurement of contact events) to eliminate reporting biases.

Another data collection after the pandemic would be interesting from several points of view: First, it might strengthen the findings on the restraining effects of social distancing on social touch. Second, long-lasting changes in individuals’ touching behavior due to the pandemic could be studied.

## 5 Conclusion

In times where touching is prohibited for the sake of protecting one’s health and the health of others, the importance of daily social touch has become apparent. The present study’s findings suggest that a decline in touch is crucial since the exchange of interpersonal social touch positively influences human well-being. While some factors, such as sharing a household, go along with a higher base in experiences of social touch, they are not sufficient to buffer the restraining effects of social distancing. Having a partner poses an exception: Being in a relationship buffered the decline of social touch in the personal context of an individual and positively influenced an individual’s well-being. Important insights can be drawn from this: In particularly stressful times like the COVID-19 pandemic, single individuals might lack the support of a partner. Therefore, care should be provided for those who are particularly vulnerable. The present research highlights the importance of regular social touch and the need to assess the long-term consequences of touch deprivation further.

## Data Availability

The raw data supporting the conclusions of this article will be made available by the authors, without undue reservation.
